# Treg increases HepG2 cell growth by RANK-RANKL pathway

**DOI:** 10.1186/2051-1426-2-S3-P240

**Published:** 2014-11-06

**Authors:** Yunbin Ye, Zhifeng Zhou, Weiwei Gu, Feng Peng, Jieyu Li

**Affiliations:** 1 Fujian Provincial Cancer Hospital, Peoples Republic of China

## Objective

In tumor microenvironment, CD4+CD25+CD127dim/- Regulatory T cells (Treg cells) belong to a group of negative regulatory cells, which play an important role in the mechanism of immune inhibition and immune escape of liver cancer [1]. In this study, we will explore the mechanism of Treg cells increasing the growth of HepG2 cells.

## Methods

Treg cells were isolated by immunomagnetic beads from peripheral blood of patients with hepatocellular carcinoma. The proliferation of HepG2 cells was determined by MTT assay. Flow cytometry was employed to detect the cell cycle distribution and the expression of RANK, RANKL on cells. The real time quantitative PCR was performed to detect the gene expression and Western blot was performed to test the level of protein. The cytokines in the cultured supernatant were tested with protein chip.

## Results

Treg cells were isolated from peripheral blood with the purity (93.83 ± 1.97) %. The proliferation rate of HepG2 cells was upregulated significantly while co-cultured with Treg cells, and the G1 ratio of HepG2 cells decreased, but S and G2 ratio increased. After co-cultured with Treg cells, the expression cyclin B and CDK1 gene of HepG2 cells were increased while the expression of P21 gene was decreased. It was also found that the Cyclin A, cyclin B and CDK1 protein level in HepG2 cells were increased while P21 was decreased. More interesting, the expression of RANKL on Treg cell was upregulated to (94.61 ± 1.56) %, and the RANK on HepG2 cell was to (96.88 ± 2.76) % in the co-culture system. The expression of IL-6, IL6R, sgp130 IL-17, TGF-β in the supernatant was increased significantly at the same time.

## Conclusion

Treg cells could change the cell cycle of HepG2 cells, and enhance the proliferation of HepG2 cells by RANK-RANKL pathway, as well as by upregulating the level of cytokines like IL-6 and IL-17. Treg might be a target to reverse the inhibitory immune microenvironment, and inhibit the growth of hepatocellular carcinoma
